# COVID-19 and Mental Health—What Do We Know So Far?

**DOI:** 10.3389/fpsyt.2020.565698

**Published:** 2020-10-26

**Authors:** Carolina Ferreira dos Santos, Maria Picó-Pérez, Pedro Morgado

**Affiliations:** ^1^Centro Hospitalar Universitário Lisboa Norte, EPE, Lisboa, Portugal; ^2^Life and Health Sciences Research Institute (ICVS), School of Medicine, University of Minho, Braga, Portugal; ^3^ICVS-3Bs PT Government Associate Laboratory, Braga/Guimarães, Portugal; ^4^2CA-Braga, Centro Clínico Académico, Hospital de Braga, Braga, Portugal

**Keywords:** COVID-19, mental health, depression, anxiety, health services

## Abstract

The coronavirus disease 2019 pandemic brought several worldwide health, social, and economic disturbances—particularly associated with the imposed confinement measures—that raised concerns about an emerging public mental health crisis. Studies investigating the early mental health impact of the pandemic on general population and vulnerable groups, such as healthcare workers, revealed a high prevalence of stress, anxiety, and depression symptoms, among others, and found several risk and protective factors. Along with these findings, the risk of substance use, suicide, domestic violence, and complicated grief may increase. We further discuss interventions that can be applied at a governmental, institutional, and individual level to minimize the mental health consequences of the pandemic, such as using telehealth to provide remote support or practicing self-care. These interventions should be maintained after the initial outbreak, as current disturbances may impact long-term well-being. We encourage the development of longitudinal studies to assess long-term adaptive responses.

## Introduction

Coronavirus disease 2019 (COVID-19) is a highly transmissible disease caused by severe acute respiratory syndrome coronavirus 2 (SARS-CoV-2), a novel coronavirus discovered in Wuhan, Hubei Province, China, in the end of 2019 (1). The severity of SARS-CoV-2 infection ranges from mild or no symptoms to severe pneumonia associated with intensive care unit admission and high mortality (2).

On March 11, 2020, COVID-19 had already spread over the five continents and was officially declared a pandemic (3); as of April 23, 2020, 2.5 million confirmed cases were reported (4), while health systems were overburdening worldwide.

Without any available cure or vaccine so far, community mitigation strategies have become essential to limit the spread of the disease and reduce the load on healthcare facilities. These strategies promote social distancing and include home quarantine, telecommuting, closure of schools and nonessential services, cancellation of events, and travel restrictions (5).

The uncertainties around the transmission pattern and incubation period of COVID-19 and its potentially serious complications—along with the social confinement measures imposed by governments, the disruption of world economies, and the overabundance of information (including false rumors) in the media—raised concerns about an emerging public mental health crisis (6).

Every day, we are confronted with new information about the current pandemic; mental health in the context of COVID-19 is being widely discussed in scientific literature and among world organizations. When discussing public mental health, it is important not only to evaluate the impact of the disease, but also to develop protocols to better handle its negative consequences. Therefore, this short narrative review aims to summarize and present relevant information on both topics; on the one hand, we review the negative psychosocial effect observed on the general population and on healthcare workers and the potential impact this pandemic could have on other vulnerable groups—people affected by COVID-19, older adults, people with mental illness, people experiencing homelessness, women suffering gender violence, racial and ethnic minorities, migrants, and sexual minorities; on the other hand, we discuss mental health interventions that can be helpful in controlling both the immediate and the long-term impact of the pandemic.

## Mental Health Impact of COVID-19 Pandemic

### On the General Population

Several Chinese institutions and research groups started investigating the generalized negative psychological effect of the pandemic early in the year. A detailed online survey conducted in the beginning of the outbreak in China found that around half of the respondents were experiencing moderate or severe psychological impact; 28.8% of respondents reported moderate to severe anxiety symptoms, 16.5% reported moderate to severe depressive symptoms, and 8.1% reported moderate to severe stress levels (7). Another cross-sectional study concluded that the prevalence of posttraumatic stress symptoms in China's province of Hubei a month after the outbreak was 7% and higher in women and in participants with poor sleep quality (8). A third study analyzed posts from Weibo, a popular Chinese social media platform, using machine-learning predictive models and found that negative emotions (e.g., anxiety, depression, indignation) and sensitivity to social risks increased after the announcement of COVID-19 on January 20, 2020, while positive emotions and life satisfaction decreased (9).

Across the world, similar results were found. A worldwide study conducted from March 29 to April 14, 2020, found a high prevalence of general psychological disturbance and posttraumatic stress and depression symptoms, with 16.2% of the participants reporting suicidal ideation (10). Three to four weeks after lockdown measures were established in Italy, the Italian population reported high levels of posttraumatic stress, depression, anxiety, insomnia, perceived stress, and adjustment disorder symptoms. In this study, several risk factors were associated with poorer mental health outcomes: being a woman, being younger, being under quarantine, having a loved one deceased by COVID-19, and experiencing stressful events (i.e., work, financial, relationship, or housing problems) related to the pandemic or lockdown measures (11). A Portuguese study also identified female sex and lower age as significant risk factors, as well as lower education levels, previously diagnosed psychological or physical conditions, and interruption of psychological support during the pandemic. Additionally, this research suggests, in line with other studies, that maintaining work, exercising, having a garden at home, and spending less time consuming COVID-19-related information in the media are protective factors against psychological symptoms (12). Optimism and social support were also found to be important resilience factors (10).

Canadian researchers have also warned us of both the protective and detrimental effect of health anxiety, which may result in either health-promoting behaviors or erratic and dangerous decisions (13).

The socioeconomic impact of the pandemic plays a key role in psychological distress. Combined with social isolation and loneliness, the resulting economic crisis, having compromised millions of jobs and income sources, is expected to increase substance use (14) and suicide rates (15, 16). The imposed lockdown has also altered family dynamics, creating challenges in sustaining the household harmony. According to UNESCO, most governments have temporarily closed educational institutions (17), and many parents are struggling with keeping their children entertained at home (18). Children's psychological well-being may be affected by the adverse consequences of school closures, including interrupted learning, lack of in-person contact with classmates and teachers, poor nutrition (children faced with economic difficulties often rely on the affordable meals provided at school), and increased exposure to violence in an abusive home (19). Reports of domestic abuse are rising worldwide (20)—stress, the disruption of social and protective networks, and decreased access to services can exacerbate the risk of violence, especially for women and children (21). Finally, with hospitals not allowing visits to admitted patients and gatherings in funerals being restricted, the occurrence of complicated grief may become more likely (22).

### On Healthcare Workers

With the increasing pressure on healthcare systems, medical workers around the world have been facing persistent psychological challenges, including high risk of infection (often derived from the lack of inadequate protection equipment), frustration, exhaustion, discrimination, isolation from their loved ones (23) or worry about infecting them (24), moral injury (25), and vicarious traumatization (26). Additionally, healthcare workers in Italy reported the emotional burden of communicating with relatives, especially when delivering bad news, as they became the only bridge between isolated patients and their families (27).

Several studies were conducted during the outbreak in China to assess the mental health of the medical staff: one study determined that several healthcare workers from a tertiary hospital were experiencing depressive symptoms and that there were no significant differences between staff in COVID-19 departments and other departments (28); a second study, also concerning medical staff from a tertiary hospital, found that the incidence of anxiety was 23.04%, the incidence of posttraumatic stress disorder was 27.39%, and that they were both higher in women and nurses (29); a third study led in multiple regions of China showed depression, anxiety, insomnia, and distress symptoms among medical workers, especially in women, nurses, and frontline workers (30); a fourth study found that the frontline medical staff were twice more likely to suffer from anxiety and depression than the nonclinical staff (31). Also, a previous review looking into the impact of the three coronavirus outbreaks on posttraumatic stress disorder symptoms in healthcare workers has identified several risk factors such as the rapidly increasing flow of critical patients requiring increased medical attention, the decision-making burden and high daily fatality rates, and the constant updates of hospital procedures following advances in knowledge about the disease, among others (32).

The psychological distress evidenced in healthcare professionals working against COVID-19 may influence their job performance, affecting their attention and decision-making ability, and may also disrupt their long-term mental health (23).

### On Other Vulnerable Groups

There are some specific groups within the general population to which we should pay particular attention.

Depressive (33) and posttraumatic stress (34) symptoms are emerging among people affected by COVID-19, who may experience additional fear of the disease's consequences, loneliness, anger (35), and social stigma (36).

Older adults, being particularly affected by the fast spread and high mortality rate of the disease, are required to be more isolated from their families and social contacts, increasing the risk of developing or worsening psychiatric symptoms and further impairing their daily functioning and cognition (37).

People with underlying mental disorders may relapse or see their preexisting condition getting worse, especially with the current difficulties in attending regular outpatient appointments and treatments. Furthermore, cognitive impairment, lower personal protection and risk awareness, and confined conditions in psychiatric wards put these patients in higher risk of SARS-CoV-2 transmission (38).

Other vulnerable groups, including people experiencing homelessness (39), women suffering gender violence (40), racial and ethnic minorities (41), migrants (42), and sexual minorities (43), may also be at higher risk of suffering from psychological distress and psychiatric disorders during the pandemic.

## Strategies and Recommendations

The abovementioned findings reflect the negative psychosocial impact of the COVID-19 outbreak and the importance of developing efficient mental health interventions at a governmental, institutional, and individual level to minimize the long-term consequences.

### Governmental Action

Along with the measures taken to prevent SARS-CoV-2 transmission, it is fundamental for governments to develop and implement well-organized, coordinated, and structured nationwide interventions to mitigate the worrying public mental health impact of COVID-19, with the support of international health authorities and the research community (6).

First, it is crucial to provide the population with accurate, transparent, and up-to-date information about the pandemic situation and related decision-making, in order to increase public awareness, to reduce stress responses and indignation (9), and to counter the spread of misinformation.

Second, governments' mental health support strategy should include integration of hospital and community facilities and systematic identification of groups at risk of psychological distress to offer them early intervention, as seen in Singapore. Timely diagnosis and intervention can be better accomplished by sensitizing and educating nonpsychiatric medical teams toward mental health assessment and techniques (44). Suicide prevention services should be reinforced to provide phone or digital assessment and interventions to those who are at risk.

Third, as suggested in recent literature (15), additional measures are vital to prevent social instability and further psychological morbidity: financial and social support should be provided for those who have lost their income sources and are facing economic difficulties; community support should be established and encouraged; governments and educational institutions should create remote alternatives to in-person classroom teaching in order to prevent the interruption of the school year; and public health authorities should develop awareness campaigns against domestic violence and substance misuse. For countries that have implemented mandatory quarantine, this decision must be regularly assessed and not be maintained for longer than is strictly necessary (45).

### The Use of Telehealth

Mental health services and practitioners are required to adapt to the new circumstances by developing ways of providing remote care to isolated patients. Nowadays, with technology, that task becomes easier. Through e-mail, phone, and video consultations, and even smartphone or online applications, telehealth can make psychological interventions widely available to the public (46, 47). Evidence shows that telemental health is particularly effective in the treatment of depression, anxiety, and posttraumatic stress disorder (47). In China, several online mental health services were developed, such as online counseling and self-help intervention systems and artificial intelligence programs (48).

Despite the huge increase of digital psychiatry during the COVID-19 pandemic, there are some concerns that should be highlighted: the use of technologies can be particularly challenging for older adults, who often lack smartphones, internet access or even the skills to reach those services (37); the number of evidence-based online interventions and applications is highly limited and there is a high risk of bias in available randomized controlled studies (49); and online interventions seem to not successfully replace face-to-face appointments (50).

The integration of technologies in psychiatry practice was significantly increased by the pandemic and hybrid interventions (combining face-to-face and online interventions) could be of critical interest in the future.

### Self-Care Practices

The role of mental health practitioners includes providing psychoeducation to their patients, informing them about common and natural stress responses and teaching them some self-care practices (51). A Nature Career Column article points out seven self-care tips to help preserve our well-being during the COVID-19 pandemic: (a) managing expectations and learning to accept that low levels of concentration, motivation, and productivity during this time are normal; (b) practicing good sleep hygiene, eating healthy, and exercising; (c) identifying negative thoughts, feelings, sensations, and actions (e.g., constantly checking the latest COVID news and data) that may contribute to distress and overwhelm; (d) creating a daily routine, separating work time from leisure time; (e) being compassionate with ourselves and others; (f) maintain social contacts, using phone and video calls; and (g) focusing on the present and on things one can control—mindfulness and meditation can be useful (52).

As previously mentioned, research showed that regular physical activity is associated with better psychological outcomes during the COVID-19 outbreak (10, 12). Simple exercises can be performed at home during daily tasks (e.g., walking in the house and to the grocery store, lifting groceries, climbing stairs), with the guidance of internet videos, TV, or mobile apps (e.g., yoga, Pilates, toning workout) or even without external tools (e.g., sit-ups, push-ups, squats) (53).

### Support for Healthcare Workers

Finally, there is an urgent need to address healthcare workers' mental health with early and adequate support measures, particularly from their peers, team leaders, and managers (25), such as normalizing stress and emotions, communicating clearly, fulfilling basic needs (including regular meals and proper rest), making working hours more flexible enabling sufficient work breaks, and providing psychological help (23, 24, 54).

Micropractices using mindfulness techniques in short periods during the work day contribute to self-awareness and can be a great tool to manage challenging emotions and thoughts in a busy environment, helping to prevent burnout. These micropractices include wellness self-checking, gratitude exercises, and diaphragmatic breathing (55).

## Discussion and Final Remarks

As the disease escalates, the fear of the unknown persists and holds its own negative consequences. Coupled with the imposed socioeconomic changes, the world population—particularly healthcare professionals and other vulnerable groups—is facing increased levels of stress, anxiety, depression, and other mental health disturbances that may impact long-term well-being, as some related conditions may even only arise later. Therefore, interventions for mental health assessment and support are essential during the current COVID-19 outbreak and also throughout the following months and even years: continued care and monitoring must be provided.

We depend on high-quality research to guide us in this battle against an unprecedented enemy and to teach us what could be done better in the future. As a final remark, we encourage researchers to gather quality data about the impact of COVID-19 and develop not only cross-sectional studies measuring immediate effects, but also longitudinal studies to assess long-term stress responses and to understand how the world is adapting.

## Data Availability Statement

The original contributions presented in the study are included in the article/supplementary material, further inquiries can be directed to the corresponding author/s.

## Author Contributions

MP-P and PM designed the work. CS performed the literature review and wrote the first draft. All authors contributed to the article and approved the submitted version.

## Conflict of Interest

The authors declare that the research was conducted in the absence of any commercial or financial relationships that could be construed as a potential conflict of interest.
